# A rare complication of systemic lupus erythematosus in a 9-year-old girl: Answers

**DOI:** 10.1007/s00467-019-04412-6

**Published:** 2019-12-10

**Authors:** Aleksandra Gliwińska, Omar Bjanid, Piotr Adamczyk, Justyna Czubilińska-Łada, Anna Dzienniak, Małgorzata Morawiecka-Pietrzak, Dagmara Roszkowska-Bjanid, Aurelia Morawiec-Knysak, Maria Szczepańska

**Affiliations:** 1 Pediatric Nephrology Ward with Dialysis Division for Children, Public Clinical Hospital No. 1 in Zabrze, Poland, ul. 3 Maja 13/15, 41-800 Zabrze, Poland; 2 Department of Pediatrics, Faculty of Medical Sciences in Zabrze, Medical University of Silesia in Katowice, Poland, ul. 3 Maja 13/15, 41-800 Zabrze, Poland; 3 Department of Pediatrics, Faculty of Medical Sciences in Katowice, Medical University of Silesia in Katowice, Poland, ul. Medyków 16, 40-752 Katowice, Poland; 4 Intensive Therapy And Neonatal Pathology Ward, Public Clinical Hospital No. 1 in Zabrze, Poland, ul. 3 Maja 13/15, 41-800 Zabrze, Poland; 5 Pediatric Endocrinology Ward, Public Clinical Hospital No. 1 in Zabrze, Poland, ul. 3 Maja 13/15, 41-800 Zabrze, Poland

**Keywords:** Systemic lupus erythematosus, Hyperferritinemia, Hemophagocytic syndrome, Macrophage activation syndrome

## Complication

Serious renal involvement in systemic diseases is common and generally constitutes a pivotal prognostic factor, making those pathology frequently seen in nephrology departments. A recent study even states that, among different medical subspecialists, nephrologists deal with the most complex patients, in terms of comorbidities and other complexity markers [2]. From this somehow eclectic nephrologist’s perspective, it seems important to be aware of and keep a high level of suspicion for rare, non-renal, but potentially devastating complications of systemic diseases, like the one highlighted in this clinical case: the secondary hemophagocytic lymphohistiocytosis (HLH). When HLH complicates a rheumatic disease, it is also referred to as macrophage activation syndrome (MAS) [3].

In HLH, a number of genetic mutations, or secondary (autoimmune, infectious, or malignant) triggers, lead to a loss of control by natural killer (NK) cells and cytotoxic lymphocytes over macrophages, which results in an excessive immune activation and uncontrolled inflammatory cytokine production by those cells. This “cytokine storm” is thought to be directly responsible for the observed extensive tissue damage and multiple organ failure. Normally, NK cells and cytotoxic lymphocytes prevent excessive macrophages and other immune cell activation, by inducing their apoptosis via a cytolytic pathway that brings to mind a lethal injection, with perforin acting like the needle, and granzyme as the poison. Primary or familial forms of HLH (pHLH) are caused by monogenic recessive mutations in genes encoding perforin (*PRF1*) and proteins that transport granzyme and perforin (*MUNC13-4*, *STX11*, and *STXBP2*). The primary disease is usually more severe than the secondary forms, begins at an earlier age, and, accordingly, is treated more aggressively [4].

**Fig. 1:**
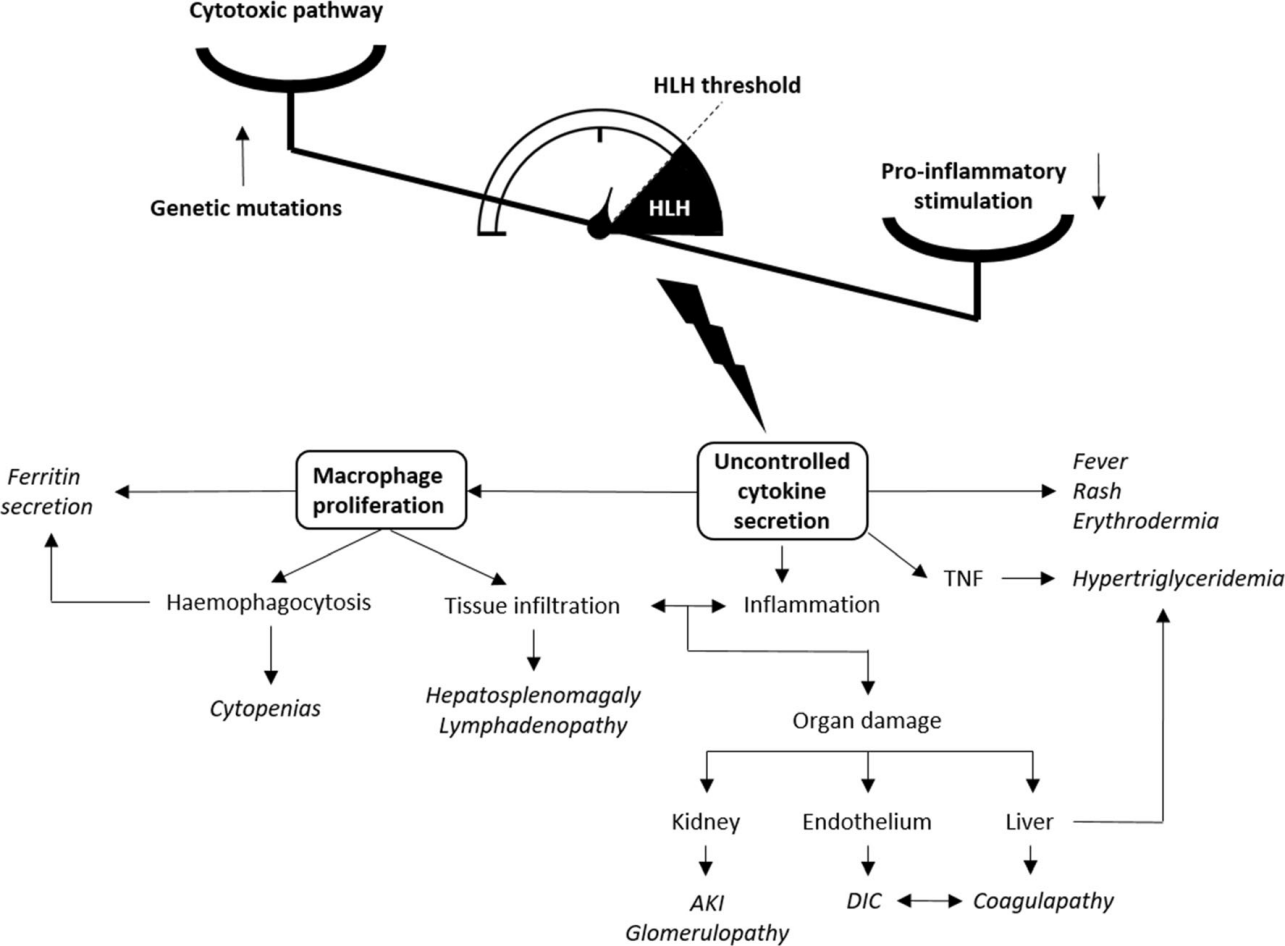
Pathogenesis and clinical features of HLH. TNF, tumor necrosis factor; AKI, acute kidney injury; DIC, disseminated intravascular coagulation

The pathogenesis of secondary HLH is more complex. Common triggers are infections, especially herpesviruses like EBV or CMV, malignancies like lymphomas and autoimmune diseases, most frequently systemic juvenile idiopathic arthritis (sJIA), and also systemic lupus erythematous (SLE) and Kawasaki disease. The incidence of MAS in patients with sJIA is relatively high and reaches 7 to 13%, whereas it remains a rare complication of SLE with an incidence of 0.9–4.6% [5]. It is not yet fully understood how a pro-inflammatory environment induces the cytolytic pathway failure to keep in check activated immune cells. It is known however that some pro-inflammatory cytokines expressed in HLH, like IL-6, decrease NK cells cytolytic activity. Moreover, increasing identification of more subtle genetic predispositions in secondary HLH, like compound heterozygous mutations, blurs the distinction between the primary/genetic and secondary/reactive forms. HLH should rather be understood as a threshold disease, where genetic factors, inflammation, infections, and immune suppression add up in different proportions in different clinical settings, to cross a point of uncontrolled pro-inflammatory amplification, and end up in a common hyperinflammatory cytokine storm pattern (Fig. 1) [6].

The clinical picture corresponds to this acute hyperinflammatory state, with unremitting fever, rash, or generalized erythema. The massive tissue infiltration caused by the proliferation of benign macrophages often presents as hemophagocytosis, hepatosplenomegaly, and lymph node enlargement. It is worth noting that hemophagocytosis, defined as the presence of fragments of blood cells within the cytoplasm of macrophages seen in bone marrow or tissues biopsies (lymph nodes, spleen, liver), while being a cardinal feature of HLH, is neither specific nor always found. It may be absent in up to 20% of children with HLH and 30% with MAS and is not therefore obligatorily required for the diagnosis [7]. Hemophagocytosis-related bone marrow involvement and consequent cytopenias are nonetheless one of the most consistent features of HLH, present in more than 80% of patients. Another key finding in HLH is the typically extremely high ferritin level, indicative of the crucial role of macrophages in hem metabolism as well as in ferritin expression and secretion [8]. Furthermore, along with interferon *γ*, interleukin-1, and other cytokines, the pro-inflammatory burst in HLH includes tumor necrosis factor (TNF), which is a potent inhibitor of lipoprotein lipase and stimulator of hepatic lipogenesis, and may be responsible for the frequently observed hypertriglyceridemia [9]. Finally, endothelium damage and liver involvement complete the typical clinical picture with DIC-like symptoms, namely bleeding diathesis, elevated d-dimers, and low fibrinogen. The latter explains the generally low ESR, contrasting with elevated CRP (Fig. 1).

## Diagnosis

**Fig. 2:**
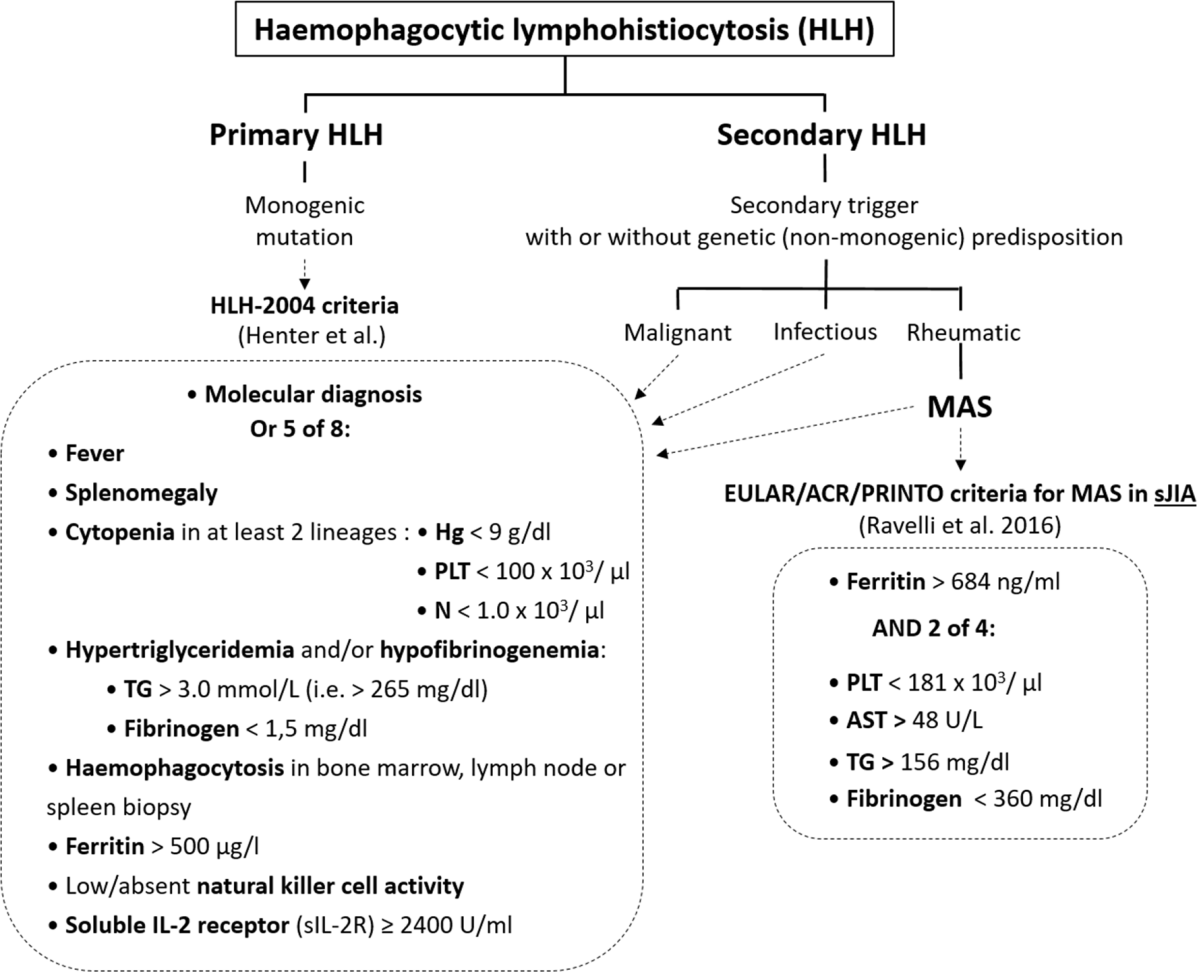
Clinical criteria of HLH. sJIA, juvenile idiopathic arthritis

The overall low incidence of the disorder, its complex pathophysiology, and the lack of unified diagnostic criteria and confusing terminology contribute to the often delayed diagnosis and treatment, which is associated with high mortality due to the aggressiveness of the clinical course. In children who develop HLH secondary to rheumatic diseases, or MAS, the mortality ranges from 8 to 40% according to different sources [10–12]. In 1991, the Histiocyte Society proposed the first diagnostic criteria for both primary and secondary HLH, which were updated later and are referred to as the HLH-2004 diagnostic and therapeutic guidelines (Fig. 2) [13, 14].

Despite the striking similarities between HLH and MAS, which made the latter being considered as a variant of secondary HLH, some differences in the clinical picture may nonetheless influence the sensitivity and specificity of the diagnostic criteria. Particularly, fever, splenomegaly, and anemia are common in systemic juvenile idiopathic arthritis (sJIA) and can hardly be considered as distinctive, whereas leukopenia is usually not present in a chronic inflammatory disease like sJIA [15]. Moreover, some of the HLH-2004 criteria, like NK cell and soluble IL-2 receptor (sIL-2R) activity, are not readily available in non-specialized centers, and the results can generally not be obtained in a timely manner to help urgent therapeutic decision-making. To cope with those issues, a group of experts led by Ravelli proposed new diagnostic criteria for MAS in sJIA in 2016 (Fig. 2) [12]. Interestingly, hemophagocytosis is not only not required but also does not figure in those criteria, which consequently have the advantage of clarity and simplicity in patients with sJIA. It would certainly be helpful if similar ones could be established for SLE MAS. Our patient met 5 out of the 8 HLH-2004 criteria (fever, splenomegaly, cytopenias, hyperferritinemia, hypertriglyceridemia, and hypofibrinogenemia), had an obvious underlying trigger (SLE), and responded well to the treatment; we therefore did not perform a bone marrow aspiration or biopsy, which obviously remain mandatory if an underlying malignancy is suspected, or in order to fulfil otherwise incomplete HLH-2004 criteria.

HLH remains challenging for the clinician in many aspects. The first one is the diagnosis and initiation of treatment despite the abovementioned diagnostic criteria, as HLH can mimic sepsis or even overlap with it when it complicates infections, for instance in patients with malignancies, immunodeficiency, or in transplant recipients. In that respect, a number of experts emphasize that an understanding of the HLH/MAS pathophysiology and its prompt recognition and treatment is crucial. If the constellation of HLH clinical signs is unfolding, treatment should not be delayed even if not all the criteria are formally fulfilled, as was the case in the presented patient [16].

## Treatment

To date, there are no controlled trials assessing the clinical management of HLH/MAS, and no definitive recommendations can be made. In the available data, based on weak evidence, small groups, or even single cases, corticosteroids remain the first-line drug. Besides the HLH-2004 protocol, based on dexamethasone, etoposide, and cyclosporine, and usually reserved for primary or critically severe HLH, treatment options in secondary HLH and MAS are usually intravenous immunoglobulins (IVIG), cyclosporine, anakinra, and cyclophosphamide. Biologicals may play an increasing role in pediatric patients. Anakinra in particular, a recombinant human interleukin-1 receptor antagonist (IL-1Ra), was recently approved by The European Medicines Agency (EMA) for the treatment of sJIA in children and may prove valuable especially in patients with sJIA MAS [17, 18].

Efforts are being made to overcome this lack of evidence-based recommendations. In an interesting ongoing collaborative initiative [19], the authors adopted a treatment algorithm in which less immunosuppressive drugs, anakinra and IVIG, are preferred in patients with serious infections and moderate HLH symptoms, whereas in the absence of serious infection or presence of critical HLH symptoms, anakinra, methylprednisolon, cyclosporine/tacrolimus, and IVIG are used. The data about treatment of SLE MAS are even scarcer. There are reports about the efficiency of cyclophosphamide, which, in the case of proliferative lupus nephritis, would seem an interesting choice, as it addresses the disease and its complication, similarly to anakinra in sJIA MAS [20, 21]. Our patient was treated with intravenous methylprednisolone at a daily dose of 10 mg/kg body weight for 4 days, then 2 mg/kg daily for 3 weeks, followed by oral treatment with prednisone (2 mg/kg daily). From the 8th day of GCS treatment, intravenous cyclophosphamide was initiated at a dose of 350 mg every 2 weeks, up to 6 doses in total. She also received hydroxychloroquine as a supplementary SLE treatment. As mentioned before, a probable early MAS relapse was observed after the kidney biopsy and was treated with 3 methylprednisolone pulses (10 mg/kg daily), while cyclophosphamide was continued according to the initially planned Euro-Lupus regimen [1]. In conclusion, HLH seems a good example of the “one size does not fit all” adage, the treatment should be individually tailored, and more specific evidence-based recommendations are needed.
